# Computer assisted orthopaedic surgery − current state and impact

**DOI:** 10.1051/sicotj/2018025

**Published:** 2018-10-01

**Authors:** Ajeya Adhikari

**Affiliations:** South West London Elective Orthopaedic Centre, London UK

The optimism of technological advances in medicine is usually tempered by the need to look at cost, benefits and the ethical and moral issues. As the technology advances benefits can seem to come in smaller and smaller increments at disproportionately large costs. The impact of new technology and its benefits is difficult to assess as it may affect wider areas like education, quality of life, societal values and efficiencies in patient journeys.

The use of computers and robots in medicine is an inevitable extension of our own lives. For the next generation of surgeons this is the only path. There are infinite variations in human anatomy and the use of technology to find a customisable solution is not science fiction but a reality that we must pursue.

As with many aspects of our lives, the use of computer technology has slowly become integral to all surgical disciplines. Orthopaedic surgery particularly lends itself to it because of the tissues we deal with. The ability to combine human acumen with technological accuracy will enhance our outcomes. The technology is now used in joint replacements, spine, tumor surgery and trauma.

As with all new technology it has gone through various phases. The advent of robot guidance in the 1990s, that of surgical navigation in the 2000s and Patient-specific instrumentation in the early 2010s was greeted with much fanfare. Device companies pushed the technology forward but the hard reality of justifying cost to the healthcare payers meant that there was a reluctance in boardrooms to finance it adequately. The technology itself proved successful in achieving what it set out to do; a highly consistent and accurate delivery of tools to help the surgeon. This was documented in well-designed studies. The early adopters continue to use the technology and there is a growing awareness among the newer surgeons about the benefits.

Computer assisted orthopaedic surgery (CAOS) can be divided into image-guided technology and landmark-based systems. The image-guided systems are virtual reality systems. MRI or CT scans are used to create a template which can then be delivered per operatively either by robots or PSI.

Navigation systems are based on reality and require the surgeon to point out landmarks at the time of surgery and require very little preoperative planning.

However, recently there has been a blurring of this division. The new generation of handheld and freestanding robots now use image guidance and navigation together to improve functionality and accuracy. In tumor surgery, there is a move to use PSI and navigation together to improve access and accuracy. Even for those who want to continue to use their conventional instruments, pinless navigation with a simple and quick workflow can be used to ensure accurate placement.

One of the issues faced by the users of this technology is that we are still using standard implants designed to be implanted using conventional instruments. This is slowly changing as custom implants are being introduced to the market.

The articles in this Special Issue attempt to bring together the current state of the technology. The case for robotics in the operating theatre is well illustrated and argued by the team from Imperial College London [1]. The planning and execution is automated using a combination of image guidance and real time data to deliver consistent results which are reproducible and not affected by human errors or fatigue. Therein lies its advantage.

The article on Robodoc from Singapore illustrates our enduring fascination with robotics. Robodoc was one of the first products in the market which spearheaded the concept and laid the groundwork for theatre set up, image guidance and delivery [2]. Though surgeons are still apprehensive of its use, further refinements and improvements continue.

The adoption of new technology and the learning curve associated with it has been well demonstrated by Jenny and Picard [3]. They have shown the benefits of using CAOS as a teaching tool. The fact that the trainee receives immediate feedback, allows him to learn but also to correct his mistake thereby reducing errors. Any reduction in the learning curve would benefit patients

The team from the Golden Jubilee Hospital in Glasgow have made the case for using the technology in areas which are not yet mainstream [4]. Hip Navigation is of particular interest as ensuring optimised placement of components in relation to each other can reduce complications like dislocations, edge loading and eventually longevity.

Lastly Hafez and Moholkar make the case for PSI which undisputedly is a convenient and efficient technique but needs further fine tuning to improve its functionality [5]. The early results were disappointing but more recent data suggests it is something which can be used in a variety of orthopaedic applications. The ability to manufacture the jigs within hospitals may well be the game changer in its adoption. The best application of this technology may be in the realms of customisation and bespoke implants.

There is no doubt that these technologies are here to stay and as they improve they will change the operating theatre of the future and the outcomes for our patients.
